# Toll-Like Receptors 2, 4, and 7, Interferon-Gamma, Interleukin 10, and Programmed Death Ligand 1 Transcripts in Leishmanin Skin Test-Positive Reactions of Ibizan Hound Dogs

**DOI:** 10.1155/2020/9602576

**Published:** 2020-03-10

**Authors:** Laura Ordeix, Sara Montserrat-Sangrà, Pamela Martínez-Orellana, Laia Solano-Gallego

**Affiliations:** ^1^Departament de Medicina i Cirurgia Animals, Facultat de Veterinària, Universitat Autònoma de Barcelona, Bellaterra 08193, Spain; ^2^Hospital Clínic Veterinari, Facultat de Veterinària, Universitat Autònoma de Barcelona, Bellaterra 08193, Spain

## Abstract

The leishmanin skin test (LST) is an *in vivo* technique commonly used to evaluate the *Leishmania*-specific cellular immune response in dogs. However, information regarding the local immune response in LST-positive reactions is scarce. We examined the pattern of toll-like receptor 2 (TLR2), TLR4, TLR7, interleukin- (IL-) 10, interferon gamma (IFN-*γ*), and (program death ligand) PD-L1 gene expression in LST-positive reactions and paired normal-looking skin of nine infected Ibizan hound dogs. Healthy skin from ten seronegative dogs from a nonendemic area was analysed as a negative control. Immune gene expressions were examined by quantitative PCR (qPCR) analysis. LST-positive reactions presented significant upregulation of TLR2, TLR4, IL-10, IFN-*γ*, and PD-L1 and downregulation of TLR7 when compared with healthy skin of seronegative control dogs from a nonendemic area. All transcripts but TLR7 were significantly higher in LST-positive reaction than in paired normal-looking skin of Ibizan hound. The expression profile of immune genes in LST-positive reactions was similar to that previously observed in clinically lesioned skin of mildly diseased dogs with papular dermatitis due to *Leishmania infantum* infection. This data provide additional support for the important role of TLRs in canine leishmaniosis.

## 1. Introduction

Canine leishmaniosis (CanL) caused by *Leishmania infantum* is a vector borne zoonotic disease endemic in many areas around the world, including many parts of the Mediterranean basin [1]. Domestic dogs are the most important mammalian reservoir for human visceral leishmaniosis, and in an endemic area, the majority of the dog population becomes infected without showing clinical evidence of disease or serum anti-*Leishmania* antibodies [2].

The outcome of *Leishmania* infection in dogs is the result of a complex immune response mounted by the host against the parasite [1, 3]. Many investigations on immune response in CanL have been focused on adaptive immune response, and the data on the importance of the innate immune responses are scarce [4]. Effective T helper 1 (Th1) cellular immunity, with the activation of macrophages by interferon gamma (IFN-*γ*) and tumour necrosis factor alpha (TNF-*α*) is associated with the elimination of intracellular amastigotes and the control of disease progression [1, 3]. Although, much of the work in CanL has focused on adaptive immune response, there is a great interest in the involvement of toll-like receptors (TLRs) in the innate immune response against *Leishmania* infection and how their expression could modulate adaptive immune response [4–7]. In fact, ligation of TLR during the early stages of infection by pathogen-associated motifs provides critical costimulatory signals for the initiation of adaptive immune responses [8]. Although numerous studies have demonstrated that engagement of individual TLR, mainly on dendritic cells, can influence CD4^+^ T cell priming and effector differentiation by skewing the Th1-Th2 balance, the role of different TLRs on adaptive immune response in *Leishmania* infection remains an unresolved issue [7]. So far, TLR2 is one of the TLRs more frequently associated with the pathogenesis of cutaneous lesions in CanL [9, 10].

There are few and poorly standardized assays to evaluate *Leishmania*-specific cellular immune responses in dogs. One of these tests is the leishmanin skin test (LST) or Montenegro's skin test [11–14]. The LST consists of the intradermal inoculation of *Leishmania* antigen and the elicitation of a delayed-type hypersensitivity (DTH) reaction in a previously infected dog [13]. Positive LST reactions in dogs are associated with mild-to-moderate disease, subclinical infections, or a positive reaction in animals after successful treatment [15–17]. On the contrary, the response is low or absent in noninfected dogs or dogs with severe disease [17].

Ibizan hounds are considered resistant dogs to *Leishmania* infection as they rarely manifest clinical disease and mount a significant cellular immune response to the infection demonstrated by a high prevalence of positive LST reactions as well as a potent *Leishmania*-specific IFN-*γ* production and low or null humoral response when compared with other breeds from the same geographical area [12, 18]. The immunohistological features of LST-positive reactions in Ibizan hound dogs have been recently described [19]. LST-positive reactions 72 hours after intradermal injection of *Leishmania* antigen were characterized by histological changes indicative of a DTH reaction similar to those findings described for human subjects [19, 20]. A moderate-to-intense, perivascular-to-interstitial dermatitis with and without panniculitis was observed. Rarely, a diffuse pattern in the deep dermis and panniculus were also observed in a few samples. There was no evidence of granuloma formation in any of the dogs studied [19]. CD3^+^ T lymphocytes were the more prominent cellular type in the dermal infiltrate. Further immunophenotyping of CD3^+^ T lymphocytes was not performed; however, marked necrosis present in all cases suggested the contribution of a cytotoxic T cell-mediated response in LST-positive reactions in resistant dogs as described for human beings [19, 20]. Moreover, TLR2 protein expression was evident by immunohistochemistry in dermal mononuclear cells [19]. Although LST is a commonly used assay to evaluate the cell-mediated immune response in dogs with leishmaniosis, to the best of the authors' knowledge, studies aimed to evaluated local immune response have not been previously published. The objective of the present study was to describe the pattern of expression of TLR2, TLR4, and TLR7, IFN-*γ*, IL-10, and PD-L1 in LST-positive reactions in Ibizan hound dogs living in a highly endemic area for leishmaniosis.

## 2. Materials and Methods

### 2.1. Dogs

Nine Ibizan hound dogs from the Island of Mallorca, Spain, with a positive LST were enrolled in this study. These dogs were also previously described in a published study aimed to evaluate the histological and immunological changes induced by intradermal inoculation of *Leishmania* antigen in resistant dogs [19]. Signalment, clinicopathological, immunological, and parasitological data from Ibizan hound dogs are summarized in Table 1. Briefly, Ibizan hounds were clinically healthy, except for three dogs that presented papulo-crusting dermatitis on the inner aspect of the pinnae suggestive of a mild form of leishmaniosis and had no clinicopathological abnormalities [1, 21]. These dogs were all infected by *L. infantum* as shown by positive LST reactions [12, 19]. Moreover, strong specific IFN-*γ* production in stimulated cultured blood was performed as described by Solano-Gallego et al. [22]. IFN-*γ* was not detected in all 9 dogs but in 6 of them [19]. In addition, blood *Leishmania* qPCR carried out as previously described [23] was negative or positive in five and four dogs, respectively [19]. All dogs were serologically negative except one (low positive) based on an in-house ELISA [19, 23].

Ten clinically healthy seronegative Beagle dogs from a nonendemic area (United Kingdom) were used as control dogs. All dogs were male and between 36 and 72 months of age. These dogs had been previously used as control subjects in other studies [5, 24, 25].

### 2.2. Skin Biopsies

Two skin samples of 6 mm were obtained from each Ibizan hound under intravenous sedation. Dogs were sedated with a combination of 0.2 ml of medetomidine (Domtor^®^, Pfizer, Madrid, Spain), 0.2 ml of butorphanol (Torbugesic Vet 10 mg/ml, Zoetis Spain, S.L., Madrid, Spain), and 0.3 ml of alphaxalone (Alfaxan 10 mg/ml®, Jurox Limited Microbial Developments, Malvern, UK). One skin sample was obtained from a LST-positive reaction at 72 hours [12] and another from normal-looking skin. Skin biopsies of LST-positive reactions were cut into two halves. One half was fixed in 10% formalin for the aforementioned immunohistological study [19] and the other half and the whole skin biopsy from normal-looking skin were submerged in RNA later (RNAlater^®^ Stabilization Solution, Ambion, Inc., Austin, Texas) stored at 4°C overnight and then kept at -80°C until used.

Moreover, skin biopsies from normal-looking skin from control dogs were used as control samples for immune gene expression. These samples were characterised by histologically normal skin and negative for *Leishmania* quantitative PCR.

### 2.3. RNA Extraction

For RNA isolation, skin samples were thawed on ice and placed in lysis solution (TRI Reagent, RiboPure™ kit, Ambion, Inc., Austin, Texas) and homogenized with a rotor-stator homogenizer (T 10 basic ULTRA-TURRAX 230 V IKA 3420000) using standard procedures. Total RNA was then isolated using the RiboPure™ kit (Ambion, Inc., Austin, Texas) under strict RNase-free conditions according to the manufacturer's protocol. In order to remove contaminating DNA, a DNase digestion step was included using TURBO DNA-free™ DNase treatment and removal reagents (Ambion, Inc., Austin, Texas) following the manufacturer's instructions. RNA concentration was determined by a Nanodrop device (Thermo Fisher Scientific Inc.). Samples had a final concentration of RNA of 34.7–251.6 ng/*μ*l. RNA integrity and quality were assessed by using an Agilent 2100 Bioanalyzer (Agilent Technologies, Santa Clara, USA). The majority of samples included in this study had an RNA integrity number value greater than 7. The recovered RNA was stored at −80°C until cDNA synthesis.

### 2.4. cDNA Synthesis

cDNA was generated from 4 *μ*l of RNA using the SuperScript™ VILO™ cDNA Synthesis kit (Invitrogen, Thermo Fisher Scientific) according to the manufacturer's instructions. cDNA was aliquoted and stored at −20°C until used for qPCR.

### 2.5. Quantitative PCR

#### 2.5.1. Immune and Reference Genes

The primers used in this study (Thermo Fisher Scientific, Carlsbad, California, USA) are listed in Table 2. Two suitable reference genes were selected as previously suggested [25–27]: Succinate dehydrogenase complex; subunit A; flavoprotein (SDHA), and similar to CG14980-PB (CG14980). PCR amplification was performed using the QuantStudio™ 12K Flex System Real-Time PCR (Thermo Fisher Scientific, Carlsbad, California, USA) using TaqMan^®^ Universal Master Mix II with UNG (Applied Biosystems, Foster City, California, USA). Plates (96 wells/plate) were filled with 0.35 *μ*l nuclease-free water (Sigma, San Luis, Missouri, USA), 7.50 *μ*l TaqMan Universal Master Mix (2×), 0.75 *μ*l TaqMan assay 20, and 6.4 *μ*l 1/5 cDNA. Plates were closed with an optical film (Applied Biosystems) centrifuged in order to mix the samples and were placed into a laboratory pipetting robot (Epmotion 5057 Liquid-handlingrobot, Eppendorf, Hamburg, Germany) to generate a 384-well plate. The 384-well plates were then transferred into a real-time PCR device. The PCR components and PCR cycler conditions were identical for the all target and reference genes. The denaturation program (95°C, 10 min), amplification, and quantification program were repeated 40 times (95°C for 15 s, 60°C for 10 s, and 72°C for 60 s) with a single fluorescence measurement. The baseline and threshold were automatically defined for the program in each run. Each sample was performed in triplicate for all the target and reference genes, and a calibrator sample (one sample from control group) was employed as control in each plate. All target genes per each dog were run on the same day and in the same plate. Data were processed while applying the relative quantification method comparable to the delta-delta-cycle threshold value (ddCt)-method. For normalization of target gene expression, the arithmetic mean of the two reference genes was taken for the calculation of a reference gene index [6]. Quantitative PCR data analyses were done by the CloudSuite software (Life Technologies™, Thermo Fisher Scientific).

#### 2.5.2. Skin Parasite Load

DNA was purified from the interphase and organic phase generated from the RNA purification process by means of QIAamp DNA Mini Kit (Qiagen, Manchester, UK) following the manufacturer's instructions with slight modifications. Briefly, 20 *μ*l of proteinase K solution and 200 *μ*l of tissue sample were used in all samples. The other steps were performed as per manufacturer's protocol. A fragment of skin from a control dog was used as a control for DNA contamination during DNA extraction. qPCR was performed with a relative quantification described elsewhere [21]. Briefly, PCR mix reaction was prepared with 4 *μ*l of DNA, 10 *μ*l of master mix (TaqMan^®^ Fast Advanced Master Mix, Thermo Fisher Scientific Inc.), 1 *μ*l of *Leishmania* primers and probes (Custom TaqMan^®^ Gene Expression Assay, ThermoFisher Scientific Inc., Waltham, USA) or 1 *μ*l of another type of assay primers and probes [Eukaryotic 18S rRNA Endogenous Control (VICTM/MGB Probe, Primer Limited, ThermoFisher Scientific Inc., Waltham, USA)] and 5 *μ*l of H_2_O. The parasite load was measured with the calculation of the delta Cq (dCq = the difference of expression between mean values of duplicate determination of *Leishmania* Cq and 18S rRNA Cq). Therefore, low or negative values of dCq represented higher parasite load than elevated dCq.

### 2.6. Statistical Analysis

Statistical analysis was performed using the blorr, generalhoslem, and Deducer packages of the R software i386 version 3.4.2 (R Development Core Team) for Windows software. Quantitative data were expressed as the geometric mean and 95% confidence interval or arithmetic mean ± standard deviation. The nonparametric Wilcoxon signed-rank test and Mann–Whitney *U* test were used to compare related and independent variables, respectively. Differences were considered significant with a 5% significance level (*p* < 0.05).

### 2.7. Ethics Approval and Consent to Participate

Dog owners were informed about the study objectives and the procedure. An informed consent was obtained from all owners for participation in the study. The procedure regarding healthy Beagle dogs followed ethical guidelines in accordance with European Union regulations and was approved by the local Ethical Review Committee from the Royal Veterinary College (University of London, UK).

## 3. Results

**Figure 1:**
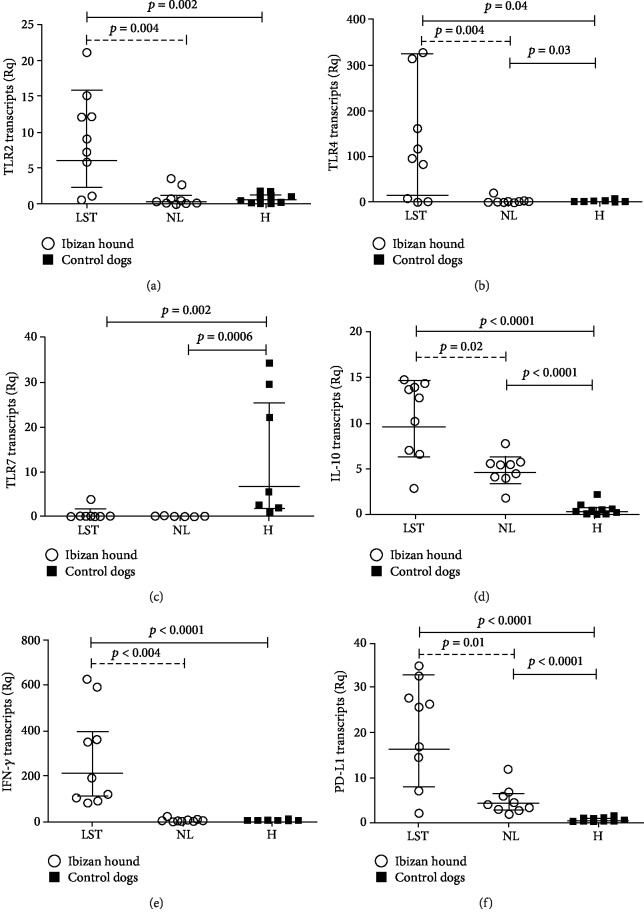
Relative quantification of the immune genes studied. (a) TLR2 transcripts. (b) TLR4 transcripts. (c) TLR7 transcripts. (d) IL-10 transcripts. (e) IFN-*γ* transcripts. (f) PD-L1 transcripts. Circles and squares represent individual data of each dog. Horizontal and vertical lines represent geometric mean and 95% confidence interval, respectively. Solid lines with *p* values: Mann–Whitney *U* test; dashed lines with *p* values: Wilcoxon signed rank test. Abbreviations: LST: leishmanin skin test; NL: normal-looking skin from Ibizan hounds; H: healthy skin from control dogs from nonendemic area; Rq: normalized relative quantification.

The expression of the immune genes studied in LST-positive reactions and paired normal-looking skin from Ibizan hound and healthy skin from seronegative control dogs from a nonendemic area is shown in Figure 1. Concisely, TLR2 (*p* = 0.002), TLR4 (*p* = 0.04), IL-10 (*p* < 0.0001), IFN-*γ* (*p* < 0.0001), and PD-L1 (*p* < 0.0001) transcripts were significantly higher in LST-positive reaction skin samples than in healthy skin from control dogs from a nonendemic area. Relative quantification of TLR7 was significantly lower in LST-positive skin than in healthy skin from seronegative control dogs from a nonendemic area (*p* = 0.002).

All the transcripts of the immune genes studied, but TLR7, were significantly higher in LST-positive reactions from Ibizan hound dogs than in paired normal-looking skin.

Only TLR4 (*p* = 0.03), IL-10 (*p* < 0.0001), and PD-L1 (*p* < 0.0001) transcripts were significantly higher in normal-looking skin from Ibizan hound dogs than in healthy skin from seronegative control dogs from nonendemic area. TLR7 (*p* = 0.0006) was downregulated in normal-looking skin of Ibizan hounds compared with healthy skin of seronegative dogs from a nonendemic area.

## 4. Discussion

To the best of the authors' knowledge, no studies have evaluated immune genes' transcripts in LST-positive reactions of dogs resistant to leishmaniosis. Moreover, similar studies have not been performed in humans or experimental animal models of leishmaniosis. Remarkably, we compared gene transcription in LST-positive reactions with paired normal-looking skin of the same dogs and healthy skin of seronegative control dogs from a nonendemic area.

LST-positive reactions were characterized by upregulation of all immune genes studied except for TLR7, when compared with normal-looking skin of Ibizan hounds and skin of seronegative control dogs from a nonendemic area. That is not an unexpected finding, given that a strong inflammatory reaction in LST was observed in these dogs based on the immunohistological study previously described elsewhere [19]. Therefore, the upregulation of immune genes in LST-positive skin reactions was the result of a severe inflammation. Moreover, parasite DNA in LST-positive reactions was higher than in paired normal-looking skin of Ibizan hound dogs and it was assumed that LST-positive reactions presented more prominent microscopic inflammatory lesions than paired normal-looking skin as described for normal-looking skin of mildly affected dogs [19, 21]. TLRs are the first to recognize *Leishmania* through various *Leishmania*-associated molecular reaction patterns leading to chemokine and cytokine expression and recruitment of inflammatory cells to the site of infection as observed in the present study [7]. In addition, these TLRs act as a connecting link between innate and adaptive immune responses [7].

Interestingly, immune gene expression profiles observed in LST-positive reactions was comparable with that observed in papular lesions of dogs with mild disease [26]. A significant upregulation of TLR2, TLR4, IL-10, IFN-*γ*, and PD-L1 was found in clinically lesioned skin of dogs with mild disease when compared with healthy skin from the same group of control dogs studied herein [24]. In that study, TLR2, TLR4, IL-10, and IFN-*γ* upregulation in clinically lesioned skin was correlated with lower disease severity [24]. Instead, downregulation of TLR2 and TLR4 and other TLRs such as TLR3 were noted in the skin and other organs in a susceptible experimental model of canine *L. infantum* infection [5]. Therefore, the immune profile detected in LST-positive reactions in Ibizan hound dogs agree with a competent immune response in these dogs. The protective role of TLR2 in several *Leishmania* species such as *L. infantum* is described elsewhere [7] and is in agreement with the present findings as well as the TLR4-related anti-*L. infantum* effects [28].

Noteworthy, relative quantification of TLR7 was significantly downregulated in LST-positive reactions from resistant Ibizan hounds when compared with healthy skin of seronegative control dogs from a nonendemic area. That was an interesting finding and in agreement with a previous study that associated TLR7 upregulation with moderate-to-severe disease [25]. In that previous study, TLR7 gene expression was significantly lower in both clinically lesioned and normal-looking skin of dogs mildly affected and with papular dermatitis than that in more severely diseased dogs or healthy skin from seronegative control dogs [25]. Moreover, TLR7 overexpression was associated with altered clinicopathological parameters suggestive of disease severity [25]. In agreement with these findings, the pathogenic role of this receptor in visceral leishmaniosis due to *L. donovani* in mice has been recently suggested [29, 30]. In this rodent model of *Leishmania* infection, an innate activation of B cells through endosomal TLRs, such as TLR7, induced cytokine (IFN type I and IL-10), and endosomal TLR expression, followed by disease exacerbation and hypergammaglobulinemia [29]. Moreover, local tissue damage mediated by persistent inflammation lead to suppression of protective T cell responses during chronic visceral leishmaniosis due to *L. donovani* in mice via TLR7 signalling [30]. Therefore, lack of TLR7 overexpression in LST-positive reactions points out a protective immune response in resistant Ibizan hounds.

Normal-looking skin of infected Ibizan hound dogs showed some changes in immune genes' transcripts when compared with healthy skin of seronegative control dogs. Noteworthy, TLR2 and IFN-*γ* expressions in normal-looking skin of infected Ibizan hound dogs were similar to healthy skin from seronegative control dogs from a nonendemic area. TLR2 overexpression is associated with an inflammatory process [5] and probably denotes lack of or less prominent microscopic inflammatory lesions in normal-looking skin of infected Ibizan hound dogs as suggested for normal-looking skin of mild diseased dogs [21]. The absence of differences in a proinflammatory cytokine such as IFN-*γ* transcription in normal-looking skin of resistant Ibizan hounds described herein is in agreement with this.

The Montenegro skin test reaction in human beings has been suggested as a surrogate of the early response to *Leishmania* infection, based on similarities in cellular immunophenotyping between acute lesions in cutaneous leishmaniosis and Montenegro skin testing of the same patient [20]. Papular dermatitis is a mild cutaneous manifestation of *L. infantum* infection classically diagnosed in young dogs, generally under one year of age, which has been clinically and experimentally associated with sand fly bite sites [31, 32]. It is likely that this is a clinical entity observed after the first contact with *L. infantum* inoculated by sand flies in an immunocompetent dog. Therefore, similarities in immune gene expression among LST-positive reactions in resistant dogs and papular dermatitis due to *Leishmania* infection [24] may reinforce the idea that papular dermatitis is an early clinical manifestation of this infection.

Some limitations in the present study should be highlighted. Firstly, the number of Ibizan hounds evaluated was low. However, this study was performed under field conditions with privately owned hunting dogs, limiting sample size. Moreover, it would also be of interest to evaluate LST-positive reactions from resistant dogs of other breeds. In addition, DTH skin reactions caused by etiologies different than *L. infantum* were not evaluated in *Leishmania*-negative Ibizan hounds in order to demonstrate that changes if immune gene transcripts were specific of *Leishmania*-associated skin inflammation or not. Unfortunately, histological examination of normal-looking skin of Ibizan hounds could not be performed. Therefore, it was not possible to definitively associate the immune genes' expression in normal-looking skin to an already present microscopic inflammation in a normal-looking skin of infected dogs [21]. Finally, normal-looking skin of noninfected Ibizan hound dogs was not evaluated. Therefore, it is not possible to demonstrate that changes in immune genes' transcripts in normal-looking skin of infected Ibizan hounds were specifically related to *Leishmania* infection.

## 5. Conclusions

The present study describes the pattern of immune gene expression in the skin of LST-positive reactions and paired normal-looking skin of Ibizan hound dogs infected by *L. infantum*, for the first time. LST-positive reactions presented significant upregulation of TLR2, TLR4, IL-10, IFN-*γ*, and PD-L1 and downregulation of TLR7 when compared with normal skin of control healthy dogs from a nonendemic area. Immune gene expression profiles in LST-positive reactions were similar to those previously observed in clinically lesioned skin of mildly diseased dogs with papular dermatitis due to *L. infantum* infection. This data provides additional support for the important role of TLRs in canine leishmaniosis.

## Figures and Tables

**Table 1:** Signalment, clinicopathological, immunological, and parasitological data of nine Ibizan hounds.

Parameters (reference intervals and units)	Ibizan hound dogs arithmetic mean ± standard deviation
Sex	2 males and 7 females
Age (months)	16 (6-84)^∗^
Creatinine (0.5 - 1.5 mg/dL)	1 ± 0.1
Urea (21.4 - 59.9 mg/dL)	33.8 ± 7.4
Total protein (5.4 - 7.1 g/dL)	6.3 ± 0.3
Albumin (2.6 - 3.3 g/dL)	3.5 ± 0.3
Albumin/globulin ratio (0.8-2)	1.2 ± 0.2
Beta globulin (0.9 - 1.6 g/dL)	1.3 ± 0.1
Gamma globulin (0.3 - 0.8 g/dL)	0.5 ± 0.1
Hematocrit (37- 55%)	49.3 ± 5.9
Hemoglobin (12 – 18 g/dL)	16.1 ± 1.8
*Leishmania infantum*-specific antibody levels (ELISA units)	18.3 ± 10.3
Blood *L. infantum*-specific IFN-*γ* (pg/ml)	3486 ± 5291.1
Blood parasite load (parasites/ml)	0.9 ± 2.2
Skin parasite load (dCq)	
LST	3.8 ± 2.6^a^
Normal-looking	8.9 ± 1.9^a^

ELISA: enzyme-linked immunosorbent assay; dCq: delta-cycle threshold (low or negative values of dCq represented high parasite density); LST: leishmanin skin test. ^a^Wilcoxon signed rank test, *Z* = −3.0267, *p* = 0.002. ^∗^Median and range.

**Table 2:** Canine reference and target genes used in this study.

Assay ID	Gene symbol	Gene name	GenBank mRNA	GenBank reference sequence	Amplicon pairwise
Cf02625049_s1	TLR2	Toll-like receptor 2	AF328930.1	NM_001005264.2	69
Cf02622203_g1	TLR4	Toll-like receptor 4	AB080363.1	NM_001002950.1	120
Cf02710573_s1	TLR7	Toll-like receptor 7	AB248956.1	NM_001048124.1	124
Cf02624265_m1	IL-10	Interleukin 10	AF328930.1	NM_001003077.1	83
Cf02623316_m1	IFN-*γ*	Interferon gamma	AF091130.1	NM_001003174.1	117
APG2FND	PD-L1	Programmed dead ligand 1	NM_001291972.1	NM_001291972.1	164
Cf02643820_m1	LOC479750	Similar to CG14980-PB	XM_536878.2	—	78
Cf02664981_m1	SDHA	Succinate dehydrogenase complex; subunit A; flavoprotein	XM_535807.2	DQ402985.1	64

## Data Availability

The datasets used and/or analysed during the current study available from the corresponding author on reasonable request.
